# Atmospheric Pressure Plasma Activation of Hydroxyapatite to Improve Fluoride Incorporation and Modulate Bacterial Biofilm

**DOI:** 10.3390/ijms222313103

**Published:** 2021-12-03

**Authors:** Maria Elena Zarif, Sașa Alexandra Yehia, Bogdan Biță, Veronica Sătulu, Sorin Vizireanu, Gheorghe Dinescu, Alina Maria Holban, Florica Marinescu, Ecaterina Andronescu, Alexandru Mihai Grumezescu, Alexandra Cătălina Bîrcă, Alexandru Titus Farcașiu

**Affiliations:** 1 Low Temperature Plasma Laboratory, National Institute for Laser, Plasma and Radiation Physics, 077125 Măgurele, Romania; maria.zarif@inflpr.ro (M.E.Z.); sasa.yehia@inflpr.ro (S.A.Y.); bogdan.bita@inflpr.ro (B.B.); veronica.satulu@inflpr.ro (V.S.); s_vizi@infim.ro (S.V.); dinescug@infim.ro (G.D.); 2 Department of Science and Engineering of Oxide Materials and Nanomaterials, Faculty of Applied Chemistry and Materials Science, University Politehnica of Bucharest, 011061 Bucharest, Romania; ecaterina.andronescu@upb.ro (E.A.); grumezescu@yahoo.com (A.M.G.); ada_birca@yahoo.com (A.C.B.); 3 Faculty of Physics, University of Bucharest, 077125 Măgurele, Romania; 4 Microbiology & Immunology Department, Faculty of Biology, University of Bucharest, 77206 Bucharest, Romania; florica.marinescu@bio.unibuc.ro; 5 Research Institute of the University of Bucharest—ICUB, University of Bucharest, 050657 Bucharest, Romania; 6 Academy of Romanian Scientists, Ilfov no. 3, 050045 Bucharest, Romania; 7 Department of Removable Prosthodontics, Faculty of Dental Medicine, Carol Davila University of Medicine and Pharmacy, 032799 Bucharest, Romania; alexandru.farcasiu@umfcd.ro

**Keywords:** dental plaque control, hydroxyapatite model, atmospheric pressure plasma, biofilm modulation, antibacterial properties, enamel fluoridation

## Abstract

Despite the technological progress of the last decade, dental caries is still the most frequent oral health threat in children and adults alike. Such a condition has multiple triggers and is caused mainly by enamel degradation under the acidic attack of microbial cells, which compose the biofilm of the dental plaque. The biofilm of the dental plaque is a multispecific microbial consortium that periodically develops on mammalian teeth. It can be partially removed through mechanical forces by individual brushing or in specialized oral care facilities. Inhibition of microbial attachment and biofilm formation, as well as methods to strengthen dental enamel to microbial attack, represent the key factors in caries prevention. The purpose of this study was to elaborate a cold plasma-based method in order to modulate microbial attachment and biofilm formation and to improve the retention of fluoride (F^−^) in an enamel-like hydroxyapatite (HAP) model sample. Our results showed improved F retention in the HAP model, which correlated with an increased antimicrobial and antibiofilm effect. The obtained cold plasma with a dual effect exhibited through biofilm modulation and enamel strengthening through fluoridation is intended for dental application, such as preventing and treating dental caries and enamel deterioration.

## 1. Introduction

Dental caries is chronic, multifactorial, and one of the most common oral diseases. It is mainly caused by tooth demineralization under the acidic attack of bacteria composing the dental plaque biofilm [1]. According to the World Health Organization [2], based on the Global Burden of Disease Study 2017 [3], 2.3 billion cases were reported for caries of permanent teeth, while 530 million cases were reported for caries of primary teeth, affecting both adults and children.

Carious lesions can be divided into non-cavitated and cavitated caries. To prevent the restorative care of the latter, non-cavitated caries must be early detected and treated using non-restorative approaches [4,5]. Fluoride administration has been the foundation of measures taken to reduce the prevalence and severity of tooth decay [6]. Fluoride can be delivered systemically and topically. The preventive effect of fluoride supplements is mainly posteruptive; however, systemic fluoride administration increases the risks of fluorosis. Therefore, topical fluoride application is preferred to the detriment of systemic fluoridation [7]. Topical fluorides are represented by home-use self-applied products (i.e., fluoride toothpaste) and professionally applied fluoride products (gel, varnish, foam), which are more concentrated and therefore are applied less frequently [8].

Cold atmospheric plasmas have several biomedical applications. In dentistry, they can be divided into indirect applications, which involve material surface modifications and direct applications, including treatments for dentin bonding, bactericidal or anticancer effects, wound healing, and aesthetics [9].

Cold atmospheric plasma treatments can be used to improve fluoride retention. Therefore, the increased uptake of fluoride by the tooth surface treated with cold atmospheric plasma [10] results in a lower acid solubility of the enamel surface [11,12]. Plasma treatments proved their antibacterial potential, recently investigated in planktonic bacteria cultures and biofilms [13]. As microbial attack and biofilm formation represent the main mechanism of caries development, atmospheric plasmas could be tailored to control the formation of dental plaque biofilm and thus inhibit caries development [14].

In order to test the hypothesis that atmospheric pressure plasma could increase enamel fluoridation and impair bacterial biofilm development, a suitable in vitro model is required. The enamel apatite is carbonated hydroxyapatite, containing different trace elements (metal cations, anionic complexes, and anions), such as Mg^2+^, K^+^, Na^+^, Ni^2+^, Cu^2+^, Zn^2+^, Mn^2+^, SO_4_^2−^, SiO_4_^4−^, Cl^−^, and F^−^, which substitute the calcium (Ca^2+^), phosphate (PO_4_^3−^), and hydroxyl (OH^−^) ions in the crystal structure [15,16]. Calcium phosphates, especially hydroxyapatite (HAP: Ca_10_(PO_4_)_6_(OH)_2_) and fluorapatite (FAP: Ca_10_(PO_4_)_6_F_2_), have biomedical applications in conservative dentistry and dental implantology, mainly due to their chemical stability and low solubility [15,17,18,19]. Moreover, the chemical and structural similarities between HAP and hard tissues, especially enamel [20], make HAP the best model for fluoridation tests.

The purpose of this study was to develop an atmospheric pressure plasma activation model to enhance fluoridation by using an HAP-based material to mimic dental enamel and the external application of a commercial fluoride gel. Additionally, the model was used to study the ability of bacteria to attach and develop monospecific biofilms on plasma-activated HAP surfaces in the presence or absence of a fluoride gel. This study approaches the main factors responsible for caries development (enamel strength modulation by improving fluoride retention and microbial attachment and biofilm development on the HAP surface). It offers innovative data for dental application and preventive oral medicine.

## 2. Results

### 2.1. Energy-Dispersive X-ray Spectroscopy

**Figure 1:**
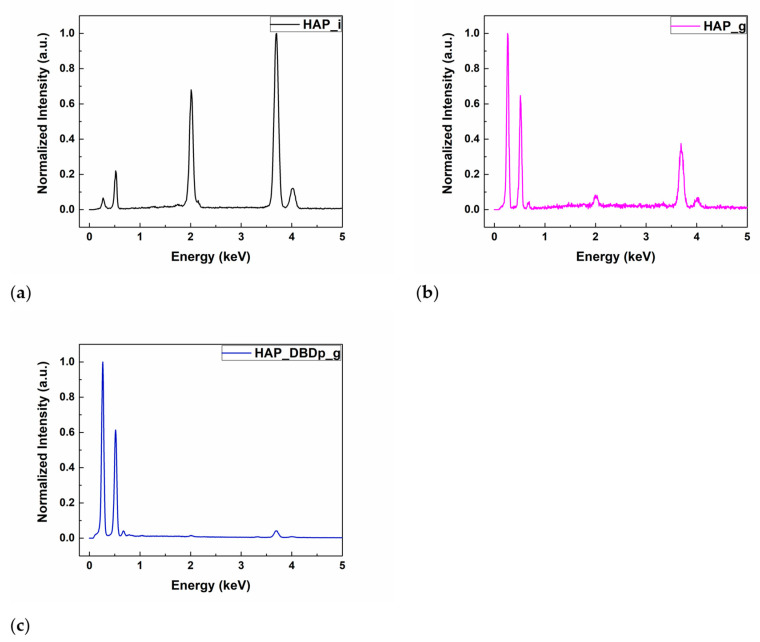
EDX spectra of (**a**) HAP_i; (**b**) HAP_g; (**c**) HAP_DBDp_g.

The energy-dispersive X-ray spectroscopy (EDX) results of the HAP samples are shown in Table 1. The identified chemical elements were C, O, Ca, P, and F. Calcium, oxygen, and phosphorus are characteristic of the chemical composition of HAP. For the gel-treated samples, without a previous plasma activation (HAP_g) or with a previous plasma activation step (HAP_DBDp_g), the presence of carbon is explained by the chemical composition of the fluoride-containing compound, while for the untreated HAP sample (HAP_i), the small amount of C (atomic %) represents residual carbon from the environment, resulting during sample manipulation. The fluorine peaks can be observed in the EDX spectra (Figure 1b,c) for HAP_g and HAP_DBDp_g samples, while the atomic percentages are presented in Table 1. For easy comparison, the EDX spectra were normalized.

It can be observed that a higher a.t. % of F was obtained for the plasma-activated HAP followed by gel application, being the most successful regarding the F incorporation (1.1%, as compared to 0.66% for HAP specimens treated with a fluoride gel in the absence of plasma activation). These results show that plasma treatments can improve the retention of F on the surface after applying an external commercial gel containing potassium fluoride.

It can be observed that a higher percentage of C is obtained for the gel-treated samples. The atomic percentage of O is higher for HAP_i and similar for the plasma-treated samples. However, the atomic percentage of Ca and P is lower for the gel-treated samples than for HAP_i. This could be explained by the surface covering of HAP with gel, which contains organic substances, and therefore, high amounts of C and O atoms. In this regard, the gel covers the HAP surface, and the atomic percentages of Ca and P on the surface, specific for the HAP chemical composition, decrease. For the gel-treated samples, the EDX results show that the surface activation using a planar Dielectric Barrier Discharge plasma source (DBDp) enhances the F retention on the HAP surface.

### 2.2. Scanning Electron Microscopy

**Figure 2:**
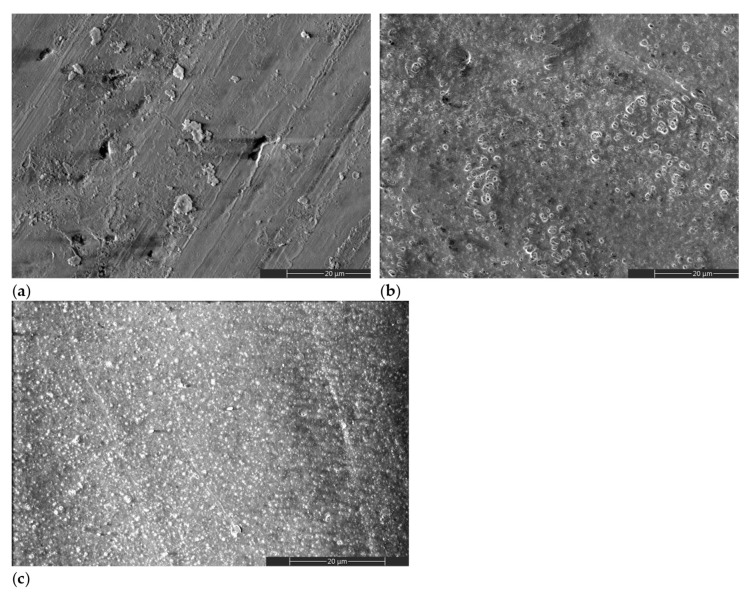
SEM images of (**a**) HAP_i; (**b**) HAP_g; (**c**) HAP_DBDp_g.

Figure 2 shows representative scanning electron microscopy (SEM) images of treated and untreated HAP samples. A different morphology can be observed for the gel-treated specimens, with (Figure 2c) or without (Figure 2b) a previous plasma activation, when compared to the initial HAP surface (Figure 2a). The surface morphology of HAP_i is obtained during the pressing step, in which surface defects can appear due to the surface asperities of the pressing mold. The other morphologies result from a dried layer of gel that remained on the surface after the removal step (Figure 2b,c).

### 2.3. X-ray Photoelectron Spectroscopy

**Figure 3:**
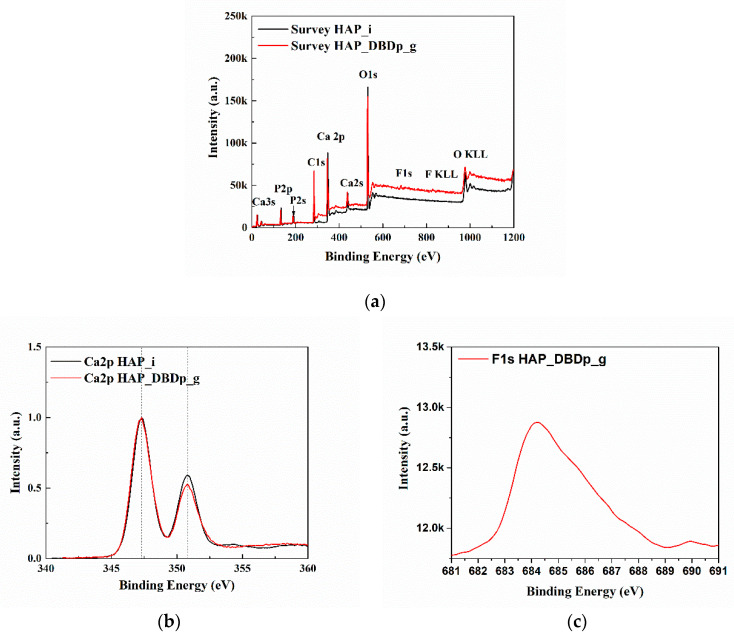
XPS spectra: (**a**) survey spectra; (**b**) high-resolution spectra of the Ca2p region; (**c**) high-resolution spectra of the F1s region.

Figure 3a presents the X-ray photoelectron spectroscopy (XPS) survey (general) spectra of HAP_i and HAP_DBDp_g. The spectra (survey and high resolution) were centered on taking into account the C1s at 284.6 eV. The identified elements are C, O, Ca, and P for all the samples. For HAP_DBDp_g, F was also identified, with a relative concentration of 1.8%. The relative atomic concentrations and the position of peaks (binding energy) characteristic for the chemical elements are shown in Table 2.

Figure 3b,c show the high-resolution spectra in the regions of Ca 2p and F 1s. For easy comparison, the spectra of Ca 2p regions were normalized and overlapped. The Ca2p spectra of HAP_i and HAP_DBDp_g present two peaks at 347.3 and 350.8 eV, usually identified as Ca 2p3/2 and Ca 2p1/2 [21]. The F1s spectrum presents one maximum at 684.2 eV (HAP_DBDp_g), which may indicate fluoride-substituted HAP [21].

### 2.4. Antibacterial Results

#### 2.4.1. Bacterial Viability in Saline Water

**Figure 4:**
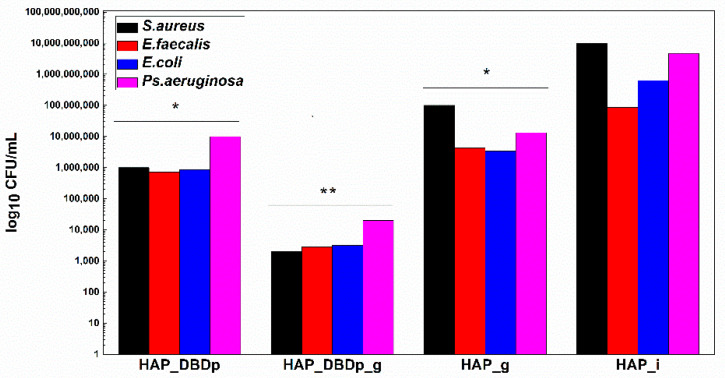
Graphic representation of microbial viability, expressed as log10 CFU (colony-forming units)/mL values, after 12 h incubation at 37 °C, on HAP specimens (one-way ANOVA, * *p* < 0.05, ** *p* < 0.001; when comparing plasma and fluoride-treated samples (HAP_DBDp, HAP_DBDp_g, and HAP_g) to untreated HAP_i sample).

In order to offer a complete image regarding the potential antimicrobial effect of the plasma-activated HAP surfaces after treatment with a fluoride-based commercial product, we assessed the viability of model microorganisms and their ability to attach and develop biofilms. The viability of the analyzed Gram-positive (*Staphylococcus aureus-S. aureus* and *Enterococcus faecalis-E. faecalis*) and Gram-negative (*Escherichia coli-E. coli* and *Pseudomonas aeruginosa*-*P. aeruginosa*) bacteria revealed that these microorganisms can survive less than 24h on the plasma-activated HAP surfaces. Viability is significantly impaired after 12 h of incubation of the microbial suspensions on the plasma-activated and fluoride gel-treated HAP samples compared to control HAP_i samples (Figure 4).

Viability is also impaired on fluoride gel-treated HAP without previous plasma activation (2 logs decrease in CFU (colony-forming units)/mL values for *S. aureus*, 1.3 logs for *E. faecalis*, 2.3 logs for *E. coli*, and 2.5 logs decrease for *P. aeruginosa*), as compared to the untreated HAP_i control.

However, there is a significantly higher difference in microbial cell viability when comparing plasma-activated samples (HAP_DBDp) to the untreated HAP_i control.

Viability on HAP_DBDp is about 4 logs decreased for *S. aureus*, 2.1 logs for *E. faecalis*, 2.9 logs for *E. coli*, and 2.7 logs decreased for *P. aeruginosa*, while the HAP_DBDp_g sample variants reduced microbial viability to up to 6.7 logs in the case of *S. aureus*, 4.5 logs in *E. faecalis*, 5.3 logs in *E. coli*, and 5.4 logs in *P. aeruginosa*, while comparing them to HAP_i samples (Figure 4).

Moreover, HAP_DBDp_g samples show a significant microbial viability decrease as compared to plasma-free fluoride gel-treated HAP_g samples (viability decrease reaches about 4.7 logs for *S. aureus*, 3.2 logs for *E. faecalis*, 3 logs for *E. coli*, and 2.9 logs for *P. aeruginosa*). The viability results reveal that HAP treatment with commercial fluoride gel may reduce bacterial viability on the HAP surface and plasma treatment increases the effect, most probably by increasing HAP fluorination.

#### 2.4.2. Planktonic Cell Growth

**Figure 5:**
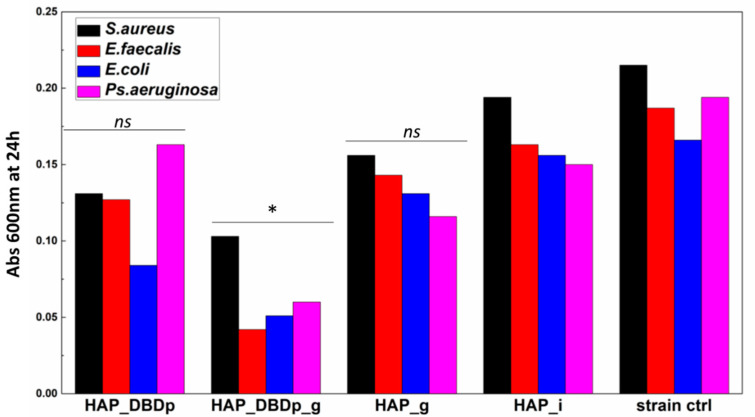
Planktonic bacterial cells grown in the presence of the developed HAP samples expressed as optical density (Abs 600 nm) values of the cultures incubated in nutritive broth for 24 h in the presence of HAP variants (one-way ANOVA, * *p* < 0.05, *ns* = not significant; when comparing plasma and fluoride-treated samples (HAP_DBDp, HAP_DBDp_g, and HAP_g) to untreated HAP_i sample).

Planktonic cell development is not significantly impacted in the presence of HAP_g and HAP_DBDp samples. One possible explanation is the fact that fluoride is not released in sufficient amounts in the culturing media for the HAP_g samples in order to significantly inhibit bacteria growth in nutritive broth (Figure 5).

Significant growth inhibition is visible in the samples encoded HAP_DBDp_g for all the analyzed bacteria strains.

These results indicate that the antibacterial properties of fluoride-treated HAP samples are enhanced by plasma activation, and this processing may improve the release of fluoride applied to the HAP material.

#### 2.4.3. Monospecific Biofilm Development

**Figure 6:**
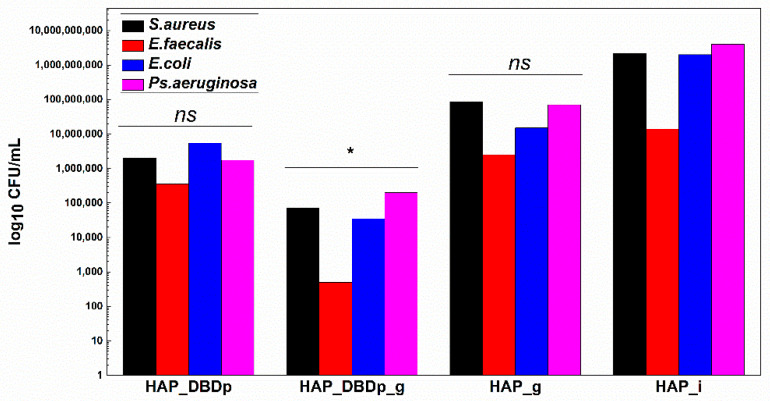
Biofilm development in the presence of HAP samples after 24 h of incubation at 37 °C. Values are expressed as log10 CFU (colony-forming units)/mL values (one-way ANOVA, * *p* < 0.05, *ns* = not significant; when comparing plasma and fluoride-treated samples (HAP_DBDp, HAP_DBDp_g, and HAP_g) to untreated HAP_i sample).

Monospecific biofilm development on the plasma-activated HAP samples treated with commercial fluoride gel was significantly reduced, as revealed in Figure 6. HAP_DBDp_g samples showed the highest biofilm development inhibition in *S. aureus*, *E. faecalis*, *E. coli*, and *P. aeruginosa*, with the CFU/mL values being reduced to up to approximately 4.5 logs in all species. Lower than the biofilm inhibition observed for HAP_DBDp_g samples, specimens encoded as HAP_DBDp also showed inhibition in biofilm formation (3 logs decrease for *S. aureus*, 1.6 logs for *E. faecalis*, 2.6 logs for *E. coli*, and 3.4 logs for *P. aeruginosa*), as compared to the untreated HAP_i control (Figure 6).

Moreover, some differences regarding biofilm inhibition were observed between HAP_g and HAP_DBDp_g, with better results for the plasma-activated samples and differences of 3.1 logs for *S. aureus*, 3.7 logs for *E. faecalis*, 2.7 logs for *E. coli*, and 2.5 logs for *P. aeruginosa*.

## 3. Discussion

This study shows the efficiency of a cold plasma activation step to improve fluoride incorporation, which was further associated with enhanced antibacterial and biofilm activities.

An enamel-like HAP model sample was used to assess the in vitro fluoridation degree using a planar DBD source. Results revealed that HAP fluoridation was higher for the combined treatment, plasma, and gel-treated surface (HAP_DBDp_g) than for the simple gel treatment (HAP_g). The incorporated F content was increased by 0.44 atomic %, as shown by EDX. The XPS results showed that in the case of HAP_DBDp_g, fluoride-substituted HAP most probably formed, with this assumption made considering the results reported in the literature regarding the binding energy for F 1s [21].

This substitution refers to the replacement of OH^−^ by F^−^, which has several effects for enamel apatite, such as increased hardness, crystal stability, and improved resistance to low pH [15]. The role of fluoride in caries prevention is well described by Buzalaf et al. [22]. In their review study [22], the authors revealed that systemic administration of fluoride is less efficient for caries prevention as compared to local treatment. Fluoride concentrations at the enamel surface are usually between 2000 and 3000 ppm, corresponding to different percentages of substitution (OH^−^ by F^−^): 6% and 8% for non-fluoridated and fluoridated areas, respectively. Moreover, the fluoride concentrations decrease with the enamel depth. These results showed that the reduction of enamel solubility will not be enhanced. It seems that above 60%, corresponding to 225,000 ppm, the solubility is low [22]. Since the 1980s, topical fluoridation has gained interest, as it was shown that that topical fluoride’s role in caries prevention is related to its effect on demineralization and remineralization at the tooth–oral fluids interface. Therefore, fluoride is involved in demineralization inhibition and remineralization enhancement. Moreover, fluoride can also affect bacterial metabolism, having an inhibitory effect. In this regard, two mechanisms were observed. The first one is related to the direct inhibition of cellular enzymes, while the second one refers to the improvement of proton permeability. Due to higher permeability, HF (hydrogen fluoride) enters through the bacterial cell membranes and dissociates in H^+^ and F^−^ in the cytoplasm [22]. Therefore, enzyme inhibition, which occurs at millimolar or micromolar levels of F^−^, has two mechanisms of action on oral bacteria: direct binding of F^−^ or HF (phosphatases, urease, enolase, heme catalase, heme peroxidase, or P-ATPase) or binding of a metal-F complex (nitrogenase or F-ATPase). In the case of proton gradient dissipation, which occurs at micromolar levels of F^−^, inhibition of export and macromolecular synthesis or cytoplasm acidification may appear. In the last case, the phosphotransferase sugar transport system, intracellular polysaccharide formation, along with glycolysis are inhibited through a mechanism in which fluoride acts as a transmembrane proton carrier [22,23,24,25].

Considering the above-mentioned results, the fluoridation degree was easily associated with the antimicrobial properties and the biofilm modulatory activity. The results showed that for plasma and gel-treated samples encoded as HAP_DBDp_g, the antibacterial effect is significantly enhanced by the plasma activation step for all bacteria models and antibacterial tests. For the bacterial viability tests, the best results were obtained for *S. aureus*, with a decrease of 6.7 logs of the microbial viability for the HAP_DBDp_g sample when compared to the untreated HAP_i control. Plasma-assisted fluoridation also showed good results regarding the biofilm inhibition for *S. aureus*, suggesting that such plasma treatment could interfere with microbial attachment and viability in a fluoride-independent manner. Moreover, the antibacterial results showed that better antibacterial effects were obtained for the plasma-assisted fluoridation (plasma activation + gel application) than for the simple fluoridation (only gel application). These results show that the increase of the fluoridation degree induces enhanced antibacterial properties, which can also be associated with a synergetic effect of the plasma activation step and the gel application.

Previous results reported in the scientific literature revealed the potential of cold atmospheric pressure plasma treatments in dentistry [26,27]. However, plasma-assisted procedures for dental fluoridation were not intensively studied. In 2018, Kim et al. showed that using a cold atmospheric plasma (CAP) device, the fluoride uptake and retention from a 1.23% acidulated P fluoride gel, along with the acid resistance, were improved on enamel specimens [10]. In 2021, Khoubrouypak et al. [28] reported the effects of helium CAP on enamel erosion when testing two fluoride varnishes (Enamelast and FluoroDose), both having concentrations of 22600 ppm F. Based on the mechanisms reported in the literature regarding the improvement of fluoride varnishes’ efficiency by CAP, two assumptions were considered at the beginning, but only the second was assumed for their study. The first one was previously reported by Kim et al. [10]. The hypothesis was based on the results reported by Laroussi and Lu in 2005 [29]. However, Kim et al. assumed that the high energy of the plasma is responsible for the separation of fluoride from fluoride products, enhancing, therefore, the replacement of hydroxyl groups by fluoride, as no data about fluoride compounds was reported by Laroussi and Lu [29]. The second hypothesis was related to improving the surface wettability after CAP treatments, reported by Chen et al. [30]. They investigated the surface modifications of different dental substrates, among which dentin and enamel were included. Different input powers and different exposure times were tested. Their results showed that for an argon plasma, the wettability of the dentin substrates increases, but with no differences in the contact angle values for different input powers or for treatments longer than 30 s. For the enamel surface, the contact angle values decreased from 24.0 ± 5.5° to 3.3 ± 1.0° after 30 s of plasma treatment. Based on the study results, Khoubrouypak et al. concluded that the CAP treatment did not significantly increase the enamel erosion resistance, which was mainly assigned to the fluoride varnish application [28].

Considering these data, we hypothesize that fluoride retention is improved by the hydrophilization of the HAP surface after an argon plasma treatment and not by the high plasma energy, which could release the fluoride from the fluoride gel. To assume the last hypothesis, the plasma treatment should be applied directly to the fluoride product. Preliminary tests (not reported here) showed that plasma treatments on fluoride gel-treated HAP samples (fluoride products were removed before the CAP treatment) determine the staining of the samples. This was associated with the chemical composition of the fluoride products, mainly organic, which plasma treatments can burn. Therefore, these experiments were not further tested.

The working gas used in the Kim et al. and Khoubrouypak et al. studies was helium, which is an expensive gas compared to argon (indicative 5.0–purity 99.999%), the gas used in our study.

The atmospheric pressure plasma activation treatment for fluoridation enhancement of HAP is a relatively new approach. This study is the first one that presents a plasma treatment model using an atmospheric pressure DBD source with an RF power supply developed for HAP fluoridation with antibiofilm activity.

Periodic fluoride treatments are recommended for patients with moderate and high caries risk. Generally, fluoride application is required every 6 months for moderate-risk patients and more frequent (between 3 and 6 months) for higher-risk patients. Fluoride gels contain higher fluoride quantities compared to fluoride varnishes. Therefore, for children younger than 6 years, fluoride varnish applications are recommended [31]. Canga et al. used a 5% sodium fluoride (22,600 ppm) compound to evaluate the fluoridation efficiency depending on the application periodicity (3, 4, and 6 months) in children aged between 6 and 10 years with permanent dentition. The best results were obtained for the 3 month periodicity, with a caries reduction of 62% [32]. Fung et al. tested the influence of silver diamine fluoride (SDF) solutions, with different concentrations (12% and 38%) and different periodicity (bi-annually and annually), in arresting carious dentine lesions in children aged between 3 and 4 years. The clinical trial showed that the best results were obtained for the 38% SDF solution applied biannually [33]. Modeer et al. previously reported that for a 5% sodium fluoride varnish applied at 3-month intervals, the progression of proximal caries was significantly reduced for patients who developed less than 8 new proximal lesions during 3 years. However, for patients with increased carious activity, the caries progression was not statistically reduced [34].

Considering these results, the improvement of fluoride retention using plasma treatments may be a solution to reduce the number of fluoride applications or to enhance the efficiency of fluoride products with lower concentrations in order to limit the risk of fluoride ingestion associated with fluoridated water, fluoride toothpaste, or products with high fluoride concentrations. However, further investigations are required to prove the time efficiency of plasma-assisted fluoridation procedures, which could reduce the number of fluoride-based treatments/year. Nevertheless, the results obtained in this study show that plasma treatments are a good option to improve fluoride retention in an enamel-like HAP model sample.

According to the World Health Organization, antimicrobial resistance (AMR) is a global concern, in the top 10 health threats worldwide. Drug-resistant pathogens are mainly caused by antimicrobials (e.g., antibiotics, antivirals, antifungals, and antiparasitics) misuse and overuse [35]. The antibiotic resistance is higher for bacterial biofilms than for planktonic cells due to different protective mechanisms [36,37,38]. Biofilms can also form in the oral cavity [39,40], causing several diseases, such as caries, endodontic infections, and periodontal diseases [41]. In this study, bacteria models, known for their ability to form biofilms [42,43], were selected for antibacterial investigations. The antibacterial properties were determined for Gram-positive (*S. aureus and E. faecalis*) and Gram-negative (*E. coli and P. aeruginosa*) bacteria models. The results revealed that CAP treatments can improve the antibacterial effects of the fluoride gel treatment. However, significant differences were observed between bacteria models for samples encoded HAP_g, HAP_DBDp, and HAP_DBDp_g, indicating therefore that both the fluoride compound and the plasma treatment induce antibacterial effects dependent on the bacterial model. In this regard, further investigations involving different fluoride products or different CAP treatments are required. Moreover, other microorganisms, such as oral streptococci (*Streptococcus mutans)* [44], oral lactobacilli (*Lactobacillus rhamnosus*) [45], or other species, such as *Bifidobacterium spp.*, *Actinomyces spp.*, or *Veillonella spp.*, associated with caries development [46] or microorganisms for which the role in caries development is still uncertain, such as *Candida albicans* [47,48,49], will be further considered as models to test the efficiency of CAP-assisted fluoridation procedures in caries prevention.

## 4. Materials and Methods

### 4.1. Hydroxyapatite

HAP powder was purchased from Sigma Aldrich. The HAP specimens were obtained using a Carver Uniaxial Press, and a pellet dies with a diameter of 6 mm. For each sample, 0.1 g of HAP powder were used. During the pressing step, a vacuum pump was connected to the pellet die to ensure the sample degassing, and a pressure of 3000 lbs (pounds) was applied. HAP samples with a diameter of 6 mm and a height of approximately 2.2 mm were obtained.

### 4.2. Fluoride-Containing Compound

A clinically used fluoride-containing gel (Fluor Protector Gel, Ivoclar Vivadent Clinical), with a concentration of 1450 ppm fluoride (F^−^), was selected for the experiments. The F source is potassium fluoride (KF).

### 4.3. Plasma Source and Plasma Treatments

**Figure 7:**
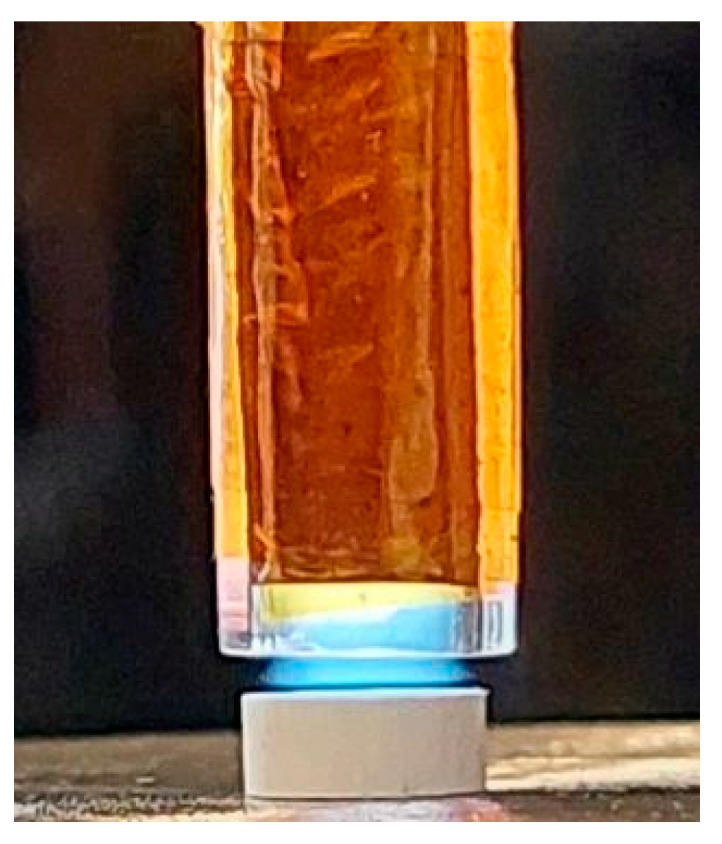
Plasma treatment on the HAP sample using the planar DBD source.

The ignition step was performed in Ar 5.0 (purity of 99.999%). The experiments were conducted using an atmospheric pressure plasma source, a planar DBD (Dielectric Barrier Discharge) source (Figure 7). The planar DBD source (DBDp) configuration consisted of a flattened glass tube (1 mm wall thickness), with rectangular external cooper electrodes (7 mm width and 70 mm length), placed as shown in Figure 7. The internal gap at the end of the DBDp source (the space delimited by the glass) is about 2 mm × 4.8 mm, leaving the plasma jet to exit from the tube (Figure 7).

The plasma source was capacitively coupled with a radiofrequency power supply (RF, 13.56 MHz). The discharges were performed with an RF generator (Cito Plus 1310-ACNA-N37A-FP; 13.56 MHz, 1000 W) purchased from COMET, Plasma Control Technologies (www.comet-pct.com, accessed on 29 November 2021).

Initial HAP samples (HAP_i) were activated by Ar plasma treatments for 3 min. The gas flow was 2000 sccm (standard cubic centimeters per minute), and the power was 20 W. The source-sample distance was set to 0.8 mm.

After plasma activation, the fluoride-containing gel was applied using cotton swabs and removed after 3 min for all samples (HAP_DBDp_g). The specimens were not rinsed after the gel removal to simulate the patient application conditions of fluoride products. For comparison, the gel was also applied on HAP samples (HAP_g) without previous plasma treatment.

### 4.4. Characterization Techniques

The surface morphology of the pressed HAP samples, before and after treatments, was examined by scanning electron microscopy (SEM) using an Apreo S ThermoFisher machine with a resolution of 0.7 nm. The chemical composition of HAP samples, before and after treatments, was determined by energy-dispersive X-ray spectroscopy (EDX) and X-ray photoelectron spectroscopy (XPS). The XPS spectra were acquired using an ESCALAB™ XI+ Spectrometer from Thermo Scientific, with a monochromatic Al Kα source at 1486.6 eV. The survey spectra were recorded with a step of 1 eV, while the high-resolution spectra were recorded with a step of 0.1 eV.

### 4.5. Antimicrobial Assay

We utilized four bacteria models with great medical relevance, able to develop biofilms on different substrata, namely Gram-positive bacteria (*Staphylococcus aureus* ATCC 25923 and *Enterococcus faecalis* ATCC 29212) and Gram-negative bacteria (*Escherichia coli* ATCC 25922 and *Pseudomonas aeruginosa* ATCC 27853). Microorganisms were maintained as glycerol stocks at −80 °C. Fresh nutritive agar (Sigma Aldrich) cultures were obtained from each microbial species, and colonies developed after 18–20 h at 37 °C were used to obtain 0.5 Mc Farland suspensions (1.5 × 10^8^ CFU (colony-forming units)/mL)) to be utilized for all further experiments [50,51].

All HAP specimens were sterilized by UV exposure (20 min on each side) before the antimicrobial tests.

#### 4.5.1. Bacterial Viability in Sterile Saline

In order to evaluate bacterial survival on the obtained HAP specimens, a volume of 50 µL of 0.5 Mc Farland suspensions was aseptically placed on each HAP specimen, and the samples were incubated in a humid chamber for up to 24 h at 37 °C. The viability of microorganisms was analyzed after 12 and 24 h of incubation by aseptically removing HAP specimens and placing each of them in 1mL of sterile saline water (0.9% NaCl prepared in distilled water and sterilized by autoclavation) in a 1.5 mL Eppendorf tube. Each Eppendorf tube was then vigorously vortexed for 20 s to detach any attached bacteria from the HAP surface, and as obtained, microbial cell suspensions were subjected to serial decimal dilutions in sterile saline. Dilutions were inoculated in triplicate on nutritive agar plates and then incubated for 24 h at 37 °C to allow the growth of viable colonies. Viable cell counts (VCCs) were performed, and CFU/mL values were calculated [52]. The most relevant results regarding viability were obtained after 12 h of incubation, while after 24 h, no viable cells were recovered from fluoride gel and plasma-treated samples (data not shown).

#### 4.5.2. Planktonic Cell Growth

The effect of the obtained materials on the growth of microorganisms in nutritive broth (planktonic cultures) was tested. A piece of sterile material was individually deposited in a well of a sterile 24-well plate. Over the deposited materials, 1 mL of liquid medium (nutritive broth, Sigma Aldrich, St. Louis, MO, USA) and subsequently 10 µL of 0.5 McFarland density microbial suspensions prepared in PBS were added. The prepared plates were incubated at 37 °C for 24 h. Then, 150 µL of the obtained microbial culture (planktonic cells) were transferred to 96-well plates, and the turbidity (optical density) of the microbial cultures (absorbance, Abs 600 nm) was measured spectrophotometrically [52].

#### 4.5.3. Monospecific Biofilm Development

The biofilm formation was analyzed in nutritive broth using a static biofilm formation model at 24 h, as previously described [53]. Briefly, HAP specimens were aseptically placed into sterile 24-well plates in 1 mL of nutritive broth (Sigma, St. Louis, MO, USA). Then, 10 µL of 0.5 McFarland suspensions were added to each well, and plates were incubated for 24 h at 37 °C. After incubation, each HAP specimen was briefly washed with 1 mL of sterile saline water in order to remove unattached cells and then transferred into 1 mL of sterile saline in an Eppendorf tube. Samples were then vigorously vortexed for 20 s and subjected to ultrasound for 10 s in order to detach bacteria included in biofilms developed on the HAP surfaces. The obtained microbial suspension was subjected to serial 10-fold dilutions, and each dilution was then inoculated on nutritive agar plates in triplicate. Inoculated plates were incubated for 24 h at 37 °C to allow colony development, which was used to obtain CFU/mL values, suggesting the biofilm-embedded viable cells recovered from the biofilms developed on each HAP sample [52,54].

### 4.6. Statistical Analysis

Biological results were analyzed using the one-way ANOVA repeated measures test. All statistical analyses were performed using GraphPad Prism Software, v. 5.03 (GraphPad Software, La Jolla, CA, USA, www.graphpad.com, Accesed on 16 November 2021).

## 5. Conclusions

This study reports on the development of an atmospheric plasma model with a dual effect, which could be utilized in dental medicine for enamel enhancement and caries prevention. Our plasma method showed improved fluoride retention in HAP enamel-like model samples, which was correlated with an improved antibacterial effect and biofilm modulatory activity. The developed model is very useful in dental medicine for studying the prevention and therapy of dental caries and could also be applied for other surfaces and medical devices.

## Figures and Tables

**Table 1:** Elemental composition (atomic %) of treated and untreated hydroxyapatite (HAP) surfaces measured by EDX.

Sample Code	Atomic %				
	C	O	Ca	P	F
HAP_i	5.49	45.63	31.88	17	-
HAP_g	56.4	38	4.41	0.53	0.66
HAP_DBDp_g	61.98	36.24	0.55	0.13	1.1

**Table 2:** XPS results of HAP_i and HAP_DBDp_g.

Sample Code	Chemical Element	Binding Energy (eV)	Relative Atomic Concentration (%)
HAP_i	C 1s	284.6	8.1
	O 1s	531.6	56.1
	Ca 2p	347.6	20.4
	P 2p	133.6	15.4
HAP_DBDp_g	C 1s	284.6	40.4
	O 1s	530.6	39.8
	Ca 2p	345.6	10.4
	P 2p	131.6	7.6
	F 1s	682.6	1.8

## Data Availability

Samples of the utilized materials are available upon request from the authors.
